# RDHCformer: Fusing ResDCN and Transformers for Fetal Head Circumference Automatic Measurement in 2D Ultrasound Images

**DOI:** 10.3389/fmed.2022.848904

**Published:** 2022-03-29

**Authors:** Chaoran Yang, Shanshan Liao, Zeyu Yang, Jiaqi Guo, Zhichao Zhang, Yingjian Yang, Yingwei Guo, Shaowei Yin, Caixia Liu, Yan Kang

**Affiliations:** ^1^College of Medicine and Biological Information Engineering, Northeastern University, Shenyang, China; ^2^Medical Device Innovation Center, Shenzhen Technology University, Shenzhen, China; ^3^Department of Obstetrics, Shengjing Hospital of China Medical University, Shenyang, China; ^4^Department of Ultrasound, Shengjing Hospital of China Medical University, Shenyang, China; ^5^Engineering Research Centre of Medical Imaging and Intelligent Analysis, Ministry of Education, Shenyang, China

**Keywords:** prenatal ultrasound, fetal head circumference, rotating object detection, transformers, convolutional neural network

## Abstract

Fetal head circumference (HC) is an important biological parameter to monitor the healthy development of the fetus. Since there are some HC measurement errors that affected by the skill and experience of the sonographers, a rapid, accurate and automatic measurement for fetal HC in prenatal ultrasound is of great significance. We proposed a new one-stage network for rotating elliptic object detection based on anchor-free method, which is also an end-to-end network for fetal HC auto-measurement that no need for any post-processing. The network structure used simple transformer structure combined with convolutional neural network (CNN) for a lightweight design, meanwhile, made full use of powerful global feature extraction ability of transformer and local feature extraction ability of CNN to extract continuous and complete skull edge information. The two complement each other for promoting detection precision of fetal HC without significantly increasing the amount of computation. In order to reduce the large variation of intersection over union (IOU) in rotating elliptic object detection caused by slight angle deviation, we used soft stage-wise regression (SSR) strategy for angle regression and added KLD that is approximate to IOU loss into total loss function. The proposed method achieved good results on the HC18 dataset to prove its effectiveness. This study is expected to help less experienced sonographers, provide help for precision medicine, and relieve the shortage of sonographers for prenatal ultrasound in worldwide.

## Introduction

Prenatal ultrasound is one of the most important examination methods during pregnancy due to its fast, low-risk and non-invasive characteristics. Fetal head circumference (HC) is one of the most essential biological indexes in accurate assessment of fetal development, which provides a method for monitoring fetal growth, estimating gestational age, and determining delivery mode. It is of paramount importance to ensure the continued wellbeing of mothers and newborns both during and after pregnancy. In prenatal ultrasound screening, the fetal head circumference is measured on standard plane of thalamus according to the obstetric ultrasound guidelines (1, 2), and the circumference of ellipse can be identified as the fetal HC since the contour of skull is similar to an ellipse. During measurement, the contour of skull is marked by sonographers, and the HC can be calculated by ellipse parameters which is obtained through fitting on post-processing software embedded in ultrasound equipment. Some semi-automated HC measurement is available on newer OB ultrasound machines, like GE Voluson E10 (SonoBiometry). The measurement results of semi-automated methods are directly affected by the accuracy in performing segmentation.

However, it is challenging for AI models to measure HC due to blurred or incomplete skull edge in ultrasound images. Accurate measurement can provide an important reference for the evaluation of fetal growth and development. Therefore, in order to improve efficiency, reliability, and reduce the workload of doctors, clinical practice puts forward high requirements for automatic segmentation (3). It is of great significance to develop an efficient and accurate method for automatic measurement of fetal HC.

**Figure 1:**
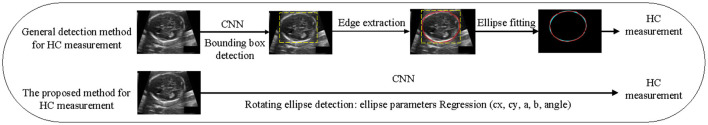
The process comparison between our method and general detection method.

In this paper, a lightweight detection network that combined with Transformer and Convolutional neural network (CNN) is proposed to detect the position of the fetal head, regress the parameters of ellipse, and then solve the head circumference value through the parameters. For automatic measurement tasks of HC, it is a one-stage network of detection. The process does not require any post-processing, such as edge extraction or ellipse fitting, and the process comparison between our method and general detection method is shown in Figure 1. This work makes the following contributions:

1. To our knowledge, our method is the first to apply the rotating ellipse detection method to the skull edge detection task. This is a one-stage network based on anchor-free method;
2. Taking Res_DCN as baseline, Deformable Convolutional Networks (DCN) combined with ResNet can learn the features of irregular boundary better and promote capability of local feature extraction. Meanwhile, powerful global feature extraction ability of Transformer is used to obtain more abundant continuous features of boundary from the global view. The proposed approach combines simple Transformer structure with CNN to obtain complete and accurate elliptical information as much as possible without significantly increasing the amount of computation;
3. Soft Stagewise Regression (SSR) strategy is used to map angle regression problems into classification problems. Firstly, the angle is roughly classified, and then the dynamic range is introduced to make every bin can do translation and scaling for fine classification. Classify the angles from coarse to fine to make angle regression accuracy higher;
4. Kullback-Leibler Divergence (KLD) loss that is similar to IOU loss is added into total loss function to solve the problem that intersection over union (IOU) between ground truth (GT) and prediction changes greatly caused by small angle deviation or center point deviation of the rotating target, as the IOU of rotate target is difficult to calculate. KLD loss can further improve the regression accuracy of elliptic parameters;
5. The proposed method gets good results compared with other existing HC measurement methods in open data set of HC18. It is noteworthy that the method is simple and efficient without requiring any post-processing.

## Related Work and Motivation

### Related Work

In the past research, many methods based on machine learning have been used to extract skull edge features, such as Haar-like features combined with different classifiers (4–9). There are also some methods based on gradient (10), threshold (11), active boundary model (12), contour fragment model (13), multi-groupfilters mixing (14) to extract features of skull region or boundary. After the skull features were extracted, different methods such as Hough transform (15) and ElliFit (16) were used to fit the elliptic skull boundary and further measure HC. Although some good results have been achieved by above methods, they all require prior knowledge or artificially designed features with poor robustness and large amount of calculation.

In recent years, CNN have been widely used in medical image segmentation (17, 18), Sinclair et al. (19) and Wu et al. (20) used the cascaded Fully Convolutional Network (FCN) to segment the skull region. U-net and its extended form have a symmetrical structure and extract rich features by using the fusion of different feature layers (21, 22). There are also some methods with different understanding of tasks, such as multi-organ segmentation (23), segmentation and regression multi-task methods (24), which are widely used in skull region or boundary segmentation. Skull boundary detection based on CNN segmentation method has excellent performance in regional segmentation, after predicting the skull region, a series of complex post-processing such as expansion, corrosion and edge extraction are carried out to obtain the skull boundary pixels, and then ellipse parameter fitting is carried out to solve HC, therefore, these methods have huge networks and cumbersome process. The measurement accuracy of head circumference depends on the segmentation result heavily, and the effect is not good for the ultrasonic image with unclear or incomplete boundary.

Object detection technology based on anchor method has good detection results for standard rectangular frame targets (25, 26), but there are no relevant studies on rotating elliptic object detection (i.e., skull edge detection task). The method based on anchor need to preset size of anchor according to IOU, and an appropriate number of anchors are selected with a certain threshold value (such as 0.5) as positive samples for regression distribution of objects. But this leads to two problems in rotating object detection: first, further aggravating the positive and negative sample imbalance. Angle prior should be added to the preset rotating anchor, doubling the number of preset anchors. In addition, rotating anchor angle slightly deviated from GT will lead to sharp decline of IOU. Second, classification is inconsistent with regression. Many studies have discussed this problem (27), that is, the classification score of predicted results is inconsistent with the positioning accuracy, so inaccurate positioning may be selected when passing the NMS stage or selecting detection results according to the classification score, while the well-positioned anchor is omitted or suppressed.

### Motivation

For skull edge detection task, due to factors such as fetuses at different gestational ages and different positions, the skull edge presents elliptic shapes of different sizes. The detection method based on Anchor needs to design sizes of different proportions according to prior, which is a very complicated process. In addition, IOU between GT and prediction changes greatly because of small angle deviation or center point deviation of the rotating target. Recently, the object detection method based on anchor-free has been greatly developed. CenterNet (28) detects the center point of the object first, and then directly regress the width and height of the object. Of course, we can directly regress a rotation angle to expand CenterNet to rotating object detection. However, the size and angle actually depend on different rotating coordinate systems, so it is difficult to directly regress parameters. To sum up, we are committed to studying a lightweight and high-precision rotating ellipse detection network for skull edge detection. The proposed method is a one-stage method based on anchor-free to solve the above problems.

## Methods

In this section, we first describe the overall architecture of the proposed method, and then explain the Gaussian distribution of GT, output maps, and KLD loss function in detail. The output maps are used to generate the oriented ellipse of the objects.

### Architecture

**Figure 2:**
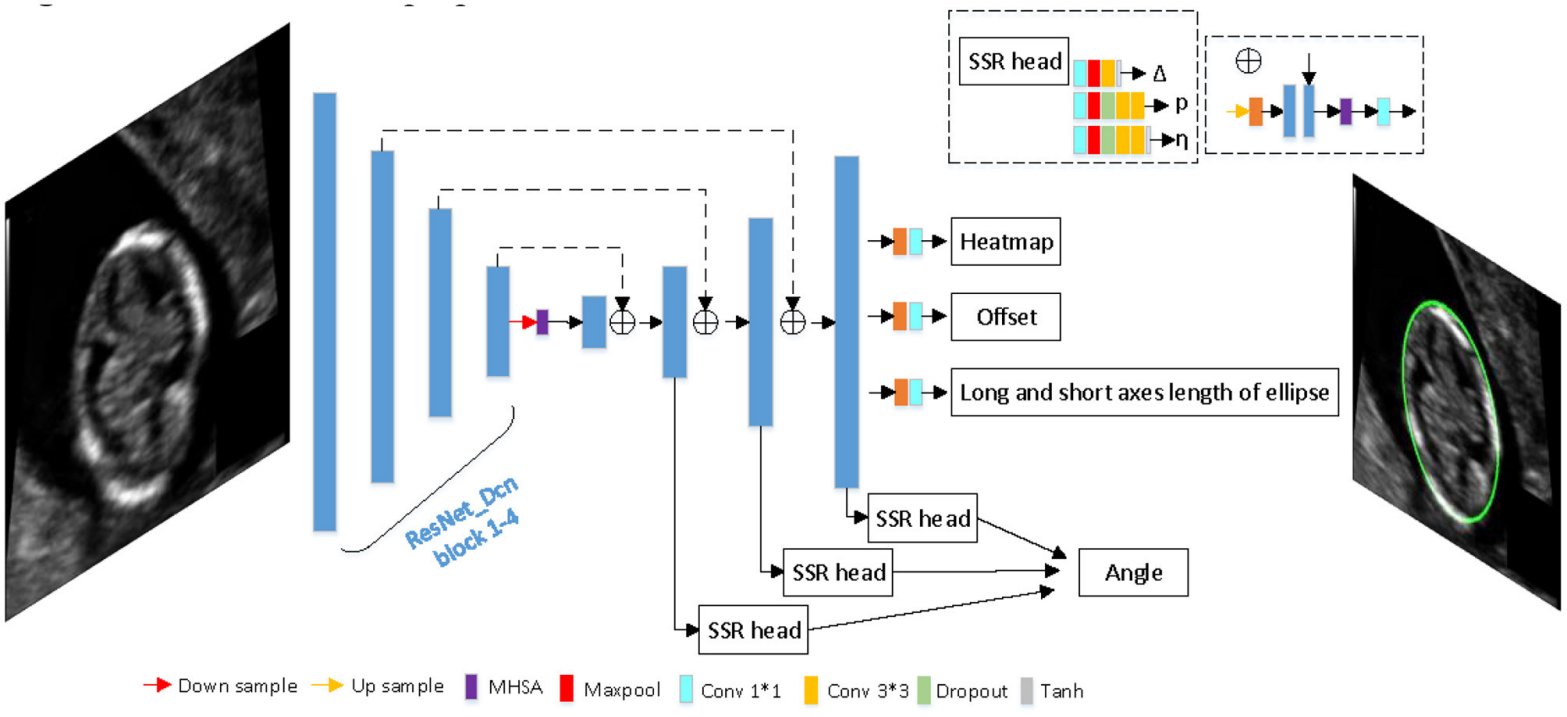
The architecture of proposed network.

**Figure 3:**
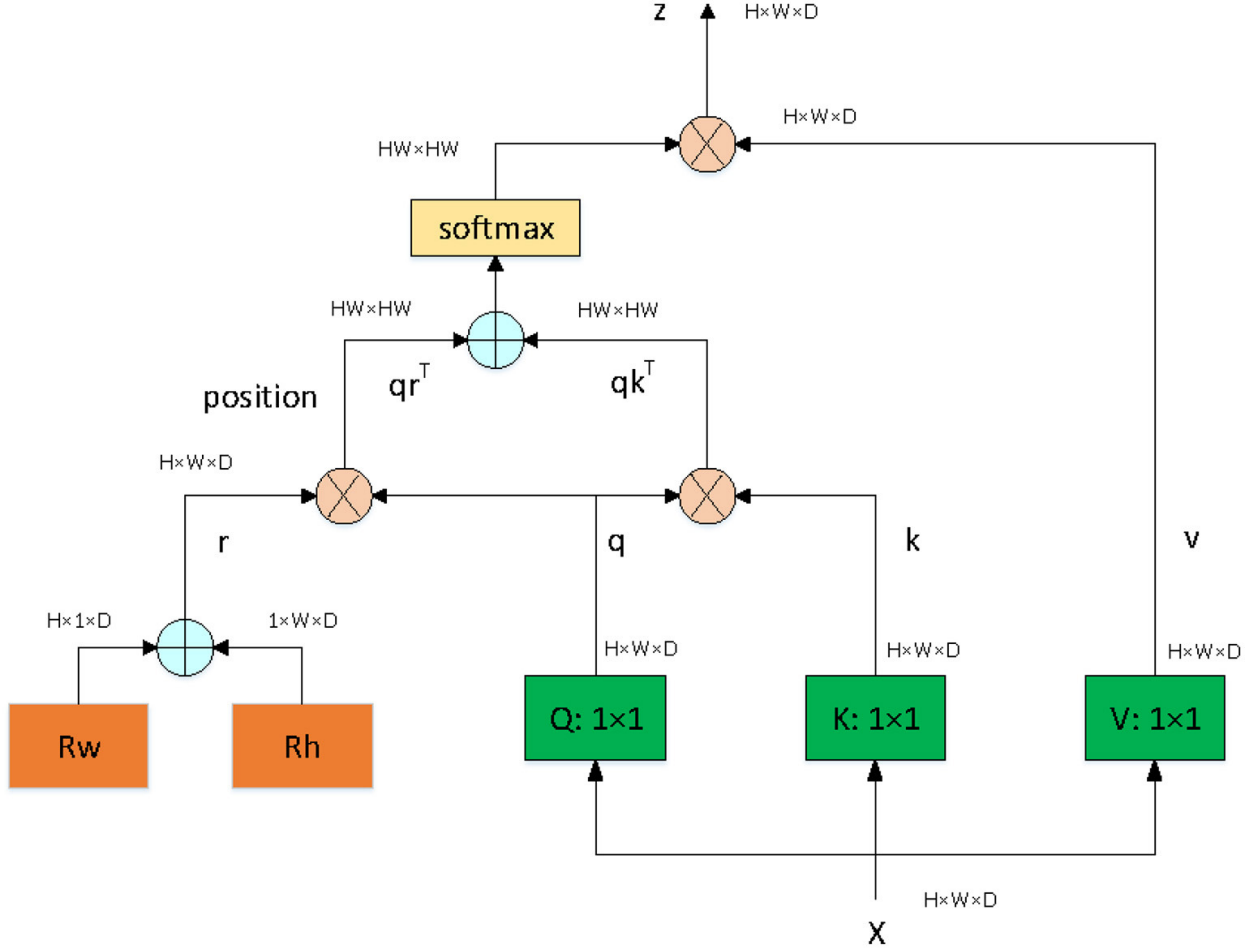
The architecture of MHSA module.

Since our goal is to build a lightweight network, we didn't choose backbone which is too complicated. The proposed network is based on an asymmetric U-shaped architecture (see Figure 2). We use the block 1–5 of ResNet_DCN as the backbone, simple Multi-head-self-attention [MHSA, see Figure 3, details in reference (29)] is used in encoder's last bottleneck module and the whole up-sampling process. Deformable convolution and self-attention mechanism are used to improve the access to local information and the continuity of irregular boundary. In decoder, output features of encoder are up-sampled to 1/4 of the input image (scale s = 4), we combine a deep layer with a shallow layer through skip connections to share both the high-level semantic information and low-level finer details. In particular, we first up-sample a deep layer to the same size of the shallow layer through bilinear interpolation. The up-sampled features map is refined through a 3 × 3 convolutional layer. The refined feature map is then concatenated with the shallow layer, followed by a 1 × 1 convolutional layer to refine the channel-wise features. In the end, four detection heads are used for ellipse parameters regression (heatmap, center offset, long and short axes of ellipse, angle).

### Gaussian Distribution of GT

**Figure 4:**
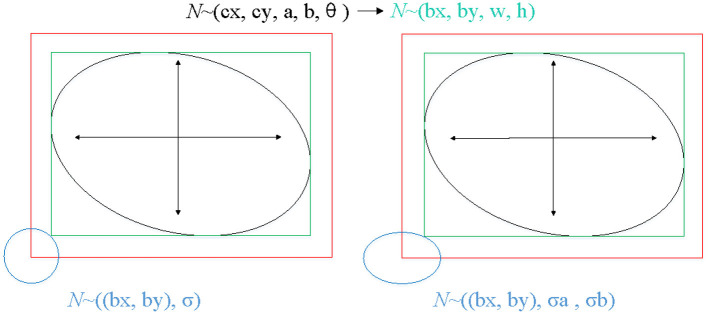
The process of mapping GT of keypoints to a 2D Gaussian distribution on the heatmap. The left shows the mapping method of reference (28), the right shows the mapping method of ours.

The proposed method locates the target based on free-anchor, we need to map GT of keypoints to a 2D Gaussian distribution on the heatmap. The mapping method in reference (28) is not friendly to targets with a large aspect ratio, especially for ellipse, so we modified the mapping method. The GT of an ellipse is (*c*_*x*_, *c*_*y*_, a, b, angle), where (*c*_*x*_, *c*_*y*_) is the center point of an oriented ellipse, a and b are long and short axes of ellipse, respectively, angle is the angle between the short axis and the vertical direction. We generate the smallest horizontal enclosing rectangular box of the ellipse (*b*_*x*_, *b*_*y*_, w, h), (*b*_*x*_, *b*_*y*_) is the center point of smallest horizontal enclosing rectangular box of the ellipse, w and h are width and height of rectangular box, respectively. We map GT (*b*_*x*_, *b*_*y*_, w, h) which can be predicted as a positive sample to 2D Gaussian distribution exp $\left(-(\frac{{\left(p_{x}-b_{x}\right)}^{2}}{2\sigma_{a}^{2}}+\frac{{\left(p_{y}-b_{y}\right)}^{2}}{2\sigma_{b}^{2}})\right)$)), where σ is a box size-adaptive standard deviation, (see Figure 4). The heatmap $P\in \;R^{c\times \frac{H}{s}\times \frac{w}{s}}$, *H* and *W* are height and width of input image respectively, *c* is set to 1 in this work.

### Output Maps

#### Heatmap

In this work, we use the heatmap $\rho \in \;R^{c\times \frac{H}{s}\times \frac{w}{s}}$ to detect the center points of arbitrarily oriented objects, where c is corresponding to one object category. The predicted heatmap value at a particular center point is regarded as the confidence of the object detection. We use the variant focal loss to train the heatmap:


(1) $$\begin{array}{l} L_{h}=-\frac{1}{N}\sum_{i}\{\begin{array}{ll} {\left(1-\rho_{i}\right)}^{\alpha }\log\left(\rho_{i}\right) & if\;P_{i}=1\\ {\left(1-P_{i}\right)}^{\beta }\rho_{i}^{\alpha }\log\left(1-\rho_{i}\right) & otherwise \end{array} \end{array}$$

where *P* and ρ refer to the ground-truth and the predicted heatmap values, *i* indexes the pixel locations on the feature, *N* is the number of objects. α and β are the hyper-parameters that control the contribution of each point. α is set to 2 and β is set to 4.

#### Center Offset

We transform GT of center point by down-sample from the input image, which is a floating-point type $C=\left(\frac{c_{x}}{s},\;\frac{c_{y}}{s}\right)$. However, the predicted center point is an integer. To compensate for the discretization error between the floating center point and the integer center point caused by the output stride, we predict an offset map $O\in R^{2\times \frac{H}{s}\times \frac{W}{s}}\;$can be defined as:


(2) $$\begin{array}{l} O=\left(\frac{c_{x}}{s}-\left\lfloor \frac{c_{x}}{s}\right\rfloor ,\frac{c_{y}}{s}-\left\lfloor \frac{c_{y}}{s}\right\rfloor \right) \end{array}$$

The offset is optimized with a smooth *L*_1_ loss:


(3) $$\begin{array}{l} L_{O}=\frac{1}{N}\overset{N}{\underset{k=1}{\sum }}Smooth\;L_{1}\left(O_{k}-o_{k}\right) \end{array}$$

where *N* is the total number of objects, *o* refers to the ground-truth offsets, *k* indexes the objects.

The smooth *L*_1_ loss can be expressed as:


(4) $$\begin{array}{l} Smooth\;L_{1}(x)=\{\begin{array}{ll} 0.5x^{2} & if\left|x\right|<1\\ \left|x\right|-0.5 & otherwise \end{array} \end{array}$$

#### Long and Short Axes of Ellipse

We regress to long and short axes of ellipse for each object, $B=\left(a,\;b\right)\in R^{2\times \frac{H}{s}\times \frac{W}{s}}$, where *a* is long axes length, *b* is short axes length. It can be optimized with a smooth *L*_1_ loss:


(5) $$\begin{array}{l} L_{O}=\frac{1}{N}\overset{N}{\underset{k=1}{\sum }}Smooth\;L_{1}\left(B_{k}-b_{k}\right) \end{array}$$

where *B* and *b* are the ground-truth and the predicted ellipse parameters, respectively.

#### Angle

**Figure 5:**
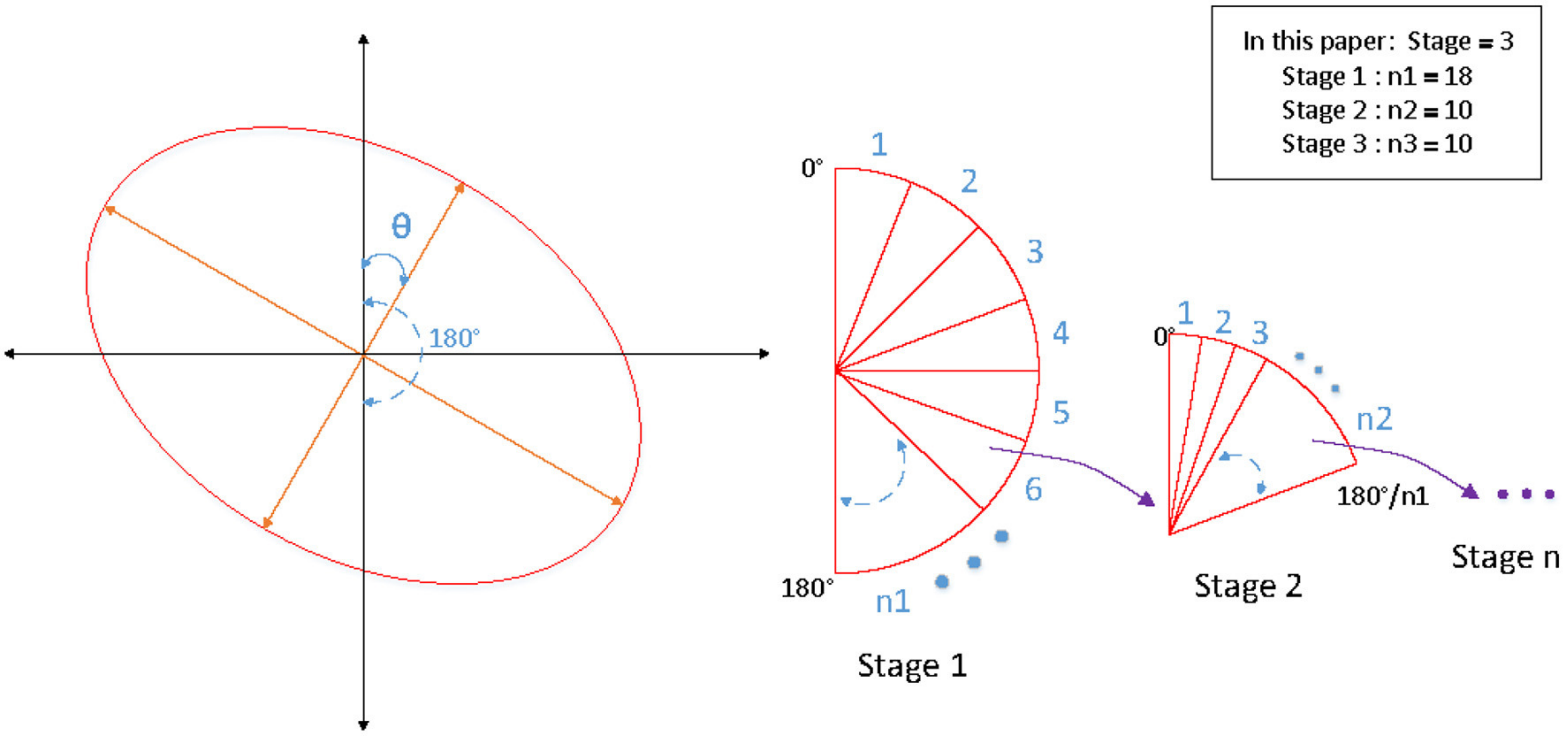
The schematic diagram of SSR strategy for angle regression.

Accurate angle regression is very important for rotating object detection, a small angle variation has marginal influence on the total loss in training, but it may induce a large IOU difference between the predicted ellipse and the ground-truth ellipse. Because of the symmetry of the ellipse, the rotation angles θ ∈ [0, 180). Soft-stagewise regression strategy is adopted for angle regression, which takes angle regression as a multi-classification task. We set it as a three-stage classification task (*S*_1_ = 18, *S*_2_ = 10, *S*_3_ = 10). In the first stage, the angle θ ∈ [0, 180) is divided into *S*_1_ parts with a span of 180 / *S*_1_. In the second stage, [0, 180 / *S*_1_] is divided into *S*_2_ parts with a span of 180 / *S*_1_ / *S*_2_. The third stage is similar, as shown in the Figure 5. In each stage, it is a multi-classification task, the sum of the probability of each class and the representative angle of the current class is taken as the final prediction value. The angle is predicted by the following formula for soft-stagewise regression:


(6) $$\begin{array}{l} \theta =\;\overset{K}{\underset{k=1}{\sum }}\overset{S_{k}-1}{\underset{i=0}{\sum }}p_{i}^{\left(k\right)}i\left(\frac{V}{\prod_{j=1}^{k}s_{j}}\right) \end{array}$$

where *V* $\in \;\left[0,\;180\right),p_{i}^{\left(k\right)}$ refers to the probability of each class for each stage, The last term in the above equation is the bin width $\omega_{k}=\frac{V}{\prod_{j=1}^{k}s_{j}}$ for the *k*-th stage and *i* is the bin index. Reference (30) introduced a dynamic range for each bin, that is, it allowed each bin to be shifted and scaled according to the input image. For adjusting the bin width ω_*k*_ at the *k*-th stage, SSR introduce a term Δ*k* to modify *s*_*k*_ into $s_{k}^{*}\;$as follows:


(7) $$\begin{array}{l} s_{k}^{*}=\;s_{k}\left(1+\Delta k\right) \end{array}$$

where Δ*k* is the output of a regression network given the input image. For shifting bins, we add an offset term η to each bin index *i*. The bin index *i* is modified as follows:


(8) $$\begin{array}{l} i=i+\eta_{i}^{\left(k\right)} \end{array}$$

Thus, the output of SSR head are $p_{i}^{\left(k\right)},\;\Delta k,and\;\eta_{i}^{\left(k\right)},\;$the angle is regressed from coarse to fine by introducing dynamic range that each bin can do translation and scaling so as to improve the precision of angle regression and reduce the error as much as possible. Angle regression can be optimized with a smooth *L*_1_ loss:


(9) $$\begin{array}{l} L_{\theta }=\frac{1}{N}\overset{N}{\underset{k=1}{\sum }}Smooth\;L_{1}\left(\theta_{k}-\theta_{k}^{*}\right) \end{array}$$

where θ_*k*_ and $\theta_{k}^{*}$ are the ground-truth and the predicted ellipse parameters, respectively.

### Kullback-Leibler Divergence Loss

**Figure 6:**
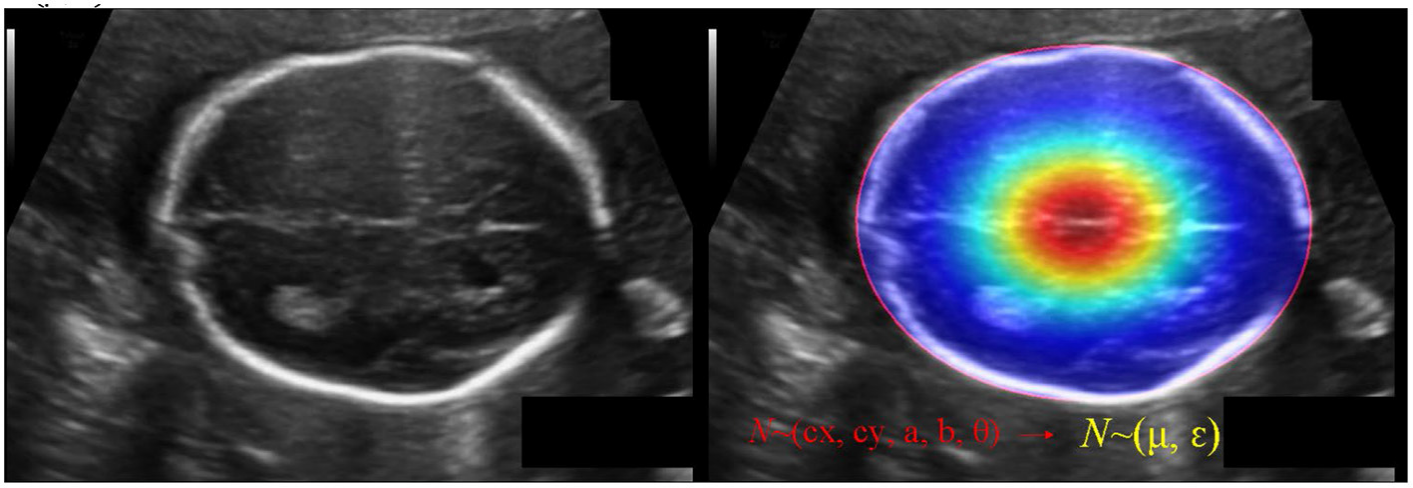
The schematic diagram of converting GT of the ellipse (*c*_*x*_, *c*_*y*_, a, b, θ) into a 2-D Gaussian *N*(μ, ε).

The IOU of rotate object is difficult to be calculated, we use KLD to measure the similarity of the two distributions to approximate the IOU. KLD loss also has some advantages when optimizing parameters. When one of the parameters is optimized, the other parameters will be used as its weight to dynamically adjust the optimization rate. In other words, the optimization of parameters is no longer independent, that is, optimizing one parameter will also promote the optimization of other parameters. The optimization of this virtuous circle is the key to KLD as an excellent rotation regression loss. Reference (31) proved its derivability and advantages. Convert GT of the ellipse (*c*_*x*_, *c*_*y*_, *a, b*, θ) into a 2-D Gaussian *N*(μ, ε), (see Figure 6). Specifically, the conversion is:


(10) $$\begin{array}{l} \mu ={\left(c_{x},\;c_{y}\right)}^{T}\\ \varepsilon^{1/2}=\left(\begin{array}{c} \frac{b}{2}\cos^{2}\theta +\frac{a}{2}\sin^{2}\theta \;\frac{b-a}{2}\cos\theta \sin\theta \\ \frac{b-a}{2}\cos\theta \sin\theta \;\frac{b}{2}\sin^{2}\theta +\frac{a}{2}\cos^{2}\theta \end{array}\right) \end{array}$$

*X*_*p*_ ~ *N*_*p*_(μ_*p*_, ε_*p*_) and *X*_*t*_ ~ *N*_*t*_(μ_*t*_, ε_*t*_), the KLD between two 2-D Gaussian is:


(11) $$\begin{array}{l} D_{kl}\left(N_{p}\|N_{t}\;\right)=\frac{1}{2}{\left(\mu_{p}-\mu_{t}\right)}^{T}\varepsilon_{t}^{-1}\left(\mu_{p}-\mu_{t}\right)+\frac{1}{2}Tr\left(\varepsilon_{t}^{-1}\varepsilon_{p}\right)\\ +\frac{1}{2}\ln\frac{\left|\varepsilon_{t}\right|}{\left|\varepsilon_{p}\right|}-1 \end{array}$$

The KLD loss is:


(12) $$\begin{array}{l} L_{reg}=1-\frac{1}{1+\log(D_{kl}\left(N_{p}\|N_{t}\right)+1)} \end{array}$$

The final regression loss is:


(13) $$\begin{array}{l} L_{total}=L_{h}+L_{O}+L_{\theta }+L_{reg} \end{array}$$

## Experiments

### Datasets and Implementation Details

Dataset is from the HC18 grand-challenge^1^ which provided 1334 2D ultrasound images from standard planes, a training set with 999 images and a test set with 335 images. Manual annotations of HC were made by senior experts. Since the data set only provides standard planes, that is to say, each image has a target, and the target accounts for a large proportion of the image. In order to balance the positive and negative samples, we used two ways to generate negative samples, one way is to remove the target in the image and fill it with surrounding information, the other way is to randomly crop the image into patches, and then resize them to the size of the network input. If the IOU with GT is <0.3, it will be considered as a negative sample. The size of each 2D ultrasound image is approximately 540 ^*^ 800 with the pixel size ranging from 0.052 to 0.6 mm. Data augmentation is essential to make model more robust. The data augmentation strategy was as follows: Rotation: rotation angle is [−30°, 30°], and the interval is 10°. Scale transformation: the scaling ratio is [0.85, 1.15], and the interval is 0.05. Gamma transformation: gamma factor is [0.5, 1.5], and the interval is 0.1. Flip: the input image is flipped randomly. After data augmentation, training set is expanded from 999 to 12,999, of which 200 are used as validation set and the rest are used as a new training set. The Stochastic Gradient Descent (SDG) optimizer is selected, the initial learning rate is set to 0.005, the momentum is 0.9, the droupout rate is 0.1, and the batchsize is set to 16. The training procedure is completed on two NVIDIA GeForce RTX 2080TI graphics cards.

### Evaluation Metrics

In order to comprehensively evaluate the performance of the model and conduct comparative analysis, regression Average Precision (AP), Mean Absolute Error (MAE), Mean Error (ME) of head circumference are adopted as the evaluation metrics of the model in this paper. HC can be calculated as follows (32):


(14) $$\begin{array}{l} HC=\pi \left[3\left(a+b\right)-\sqrt{\left(3a+b\right)\left(a+3b\right)}\right] \end{array}$$

where a and b are parameters of semi-long axis and semi-short axis of the ellipse. Mean Absolute Error of fetal HC is defined as:


(15) $$\begin{array}{l} MAE_{hc}=\frac{1}{N}\overset{N}{\underset{i=1}{\sum }}|\hat{HC_{i}}-HC_{i}| \end{array}$$

Mean Error of fetal HC is defined as:


(16) $$\begin{array}{l} ME_{hc}=\frac{1}{N}\overset{N}{\underset{i=1}{\sum }}\left(\hat{HC_{i}}-HC_{i}\right) \end{array}$$

Where $\hat{HC}$ and *HC* denote the HC measured by the proposed method and the real value of fetal HC, respectively.

Average Precision is a commonly used evaluation metric in object detection that obtained by calculating the area of Precision-Recall curve.

## Results

**Figure 7:**
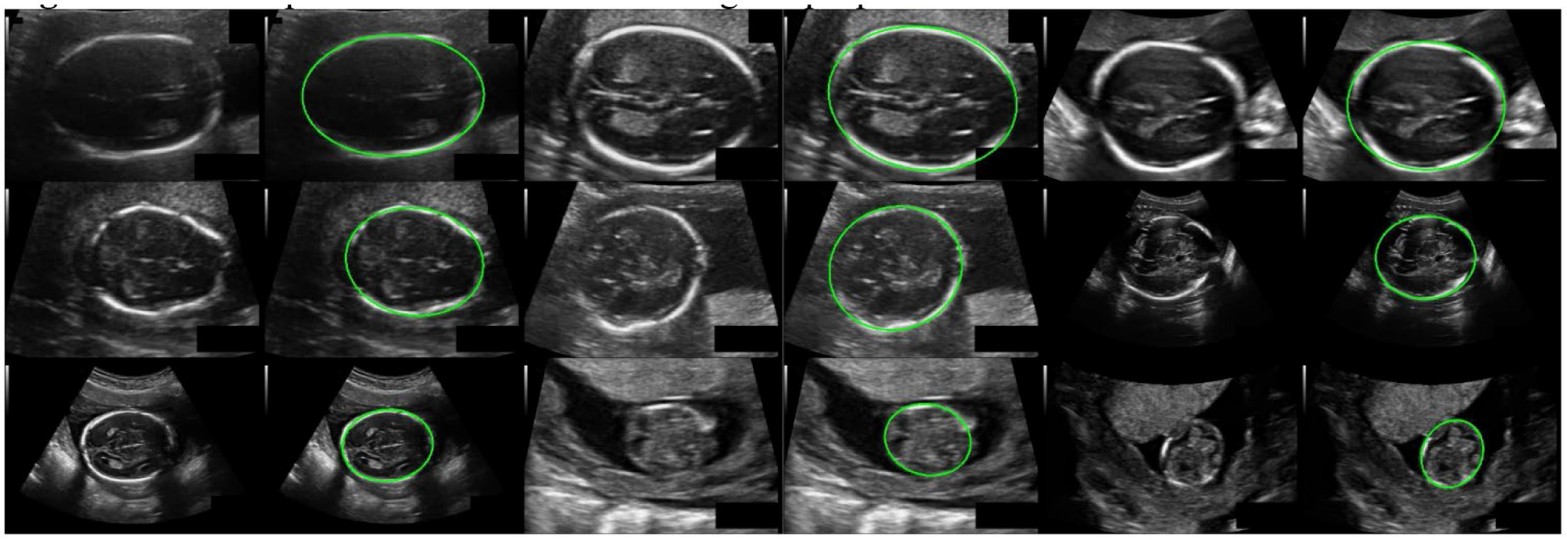
Some examples of detection results using the proposed method.

Our method has achieved good results on HC18 dataset, Average precision (AP) is 84.45%. MAE ± std (mm) is 1.97±1.89, ME ± std (mm) is 0.11±2.71, the parameter size of the proposed model is 24.31M. Table 1 shows the comparison results between our method and other common methods base on segmentation algorithm. It can be seen that our method has achieved good skull edge detection results without significantly increasing the amount of model parameters, and it can be comparable to the state-of-the-art method. It is worth noting that our method is simple and efficient. Unlike methods based on segmentation algorithm, our method do not need any complicated post-processing, which is an end-to-end network strictly for head circumference detection task. There are some examples of detection results in Figure 7.

**Table 1:** The comparison results between our method and other common methods base on segmentation algorithm.

**Model**	**MAE ±std (mm)**	**ME ±std (mm)**	**Params (M)**	**AP**
U-Net (33)	2.36 ± 5.60	0.41 ± 2.91	31.042	-
U-Net++ (34)	2.29 ± 2.33	0.27 ± 2.73	9.163	-
CE-Net (35)	2.24 ± 2.28	0.16 ± 2.12	29.003	-
SE-Unet (36)	2.27 ± 3.61	0.09 ± 2.33	**-**	-
HC18 challenge best	**1.72** **±1.60**	**0.04** **±2.35**	**-**	-
Ours	1.97 ± 1.89	0.11 ± 2.71	**24.31**	**84.45**

*MAE, Mean Absolute Error; ME, Mean Error; std, standard deviation. Param, the size of model parameters; M, Mbyte; AP, Average precision. Bold values represent the best value of each indicator*.

### Ablation Analysis

Some ablation experiments were conducted to prove the effectiveness of each module design in our algorithm. Taking Res_DCN-50 as the backbone as an example, the experimental results are as shown in Table 2. It can be seen that using normal Smooth L1 function and without MHSA module achieved the AP: 77.33%, while adding the MHSA module AP: 81.83%, increased 4.5%, it indicated that the MHSA module has a significant improvement for the task. With the addition of MHSA module, AP was increased by 1.42-83.25% by using SSR detection head for angle, then after adding KLD Loss, AP was increased by 1.2-84.45% further, at this time, compared with no MHSA module AP: 81.71%, it is an increase of 2.74%. This indicated that the excellent global feature extraction ability of MHSA module improves the model's ability to extract skull edge continuity features, it is helpful for each module of the network.

**Table 2:** Ablation experiments results.

**Backbone**	**Method**	**AP (with MHSA/ not)**
Res_DCN-50	Smooth L1 (center, a, b, angle)	81.83/77.33
	Smooth L1 (center, a, b) + SSR (angle)	83.25/79.97
	Smooth L1 (center, a, b) + SSR (angle) + KLD	**84.45**/81.71

*Comparison of Res_DCN-50 with or without MHSA module, SSR, and KLD loss. Bold values represent the best value of each indicator*.

### Consistency Analysis

**Figure 8:**
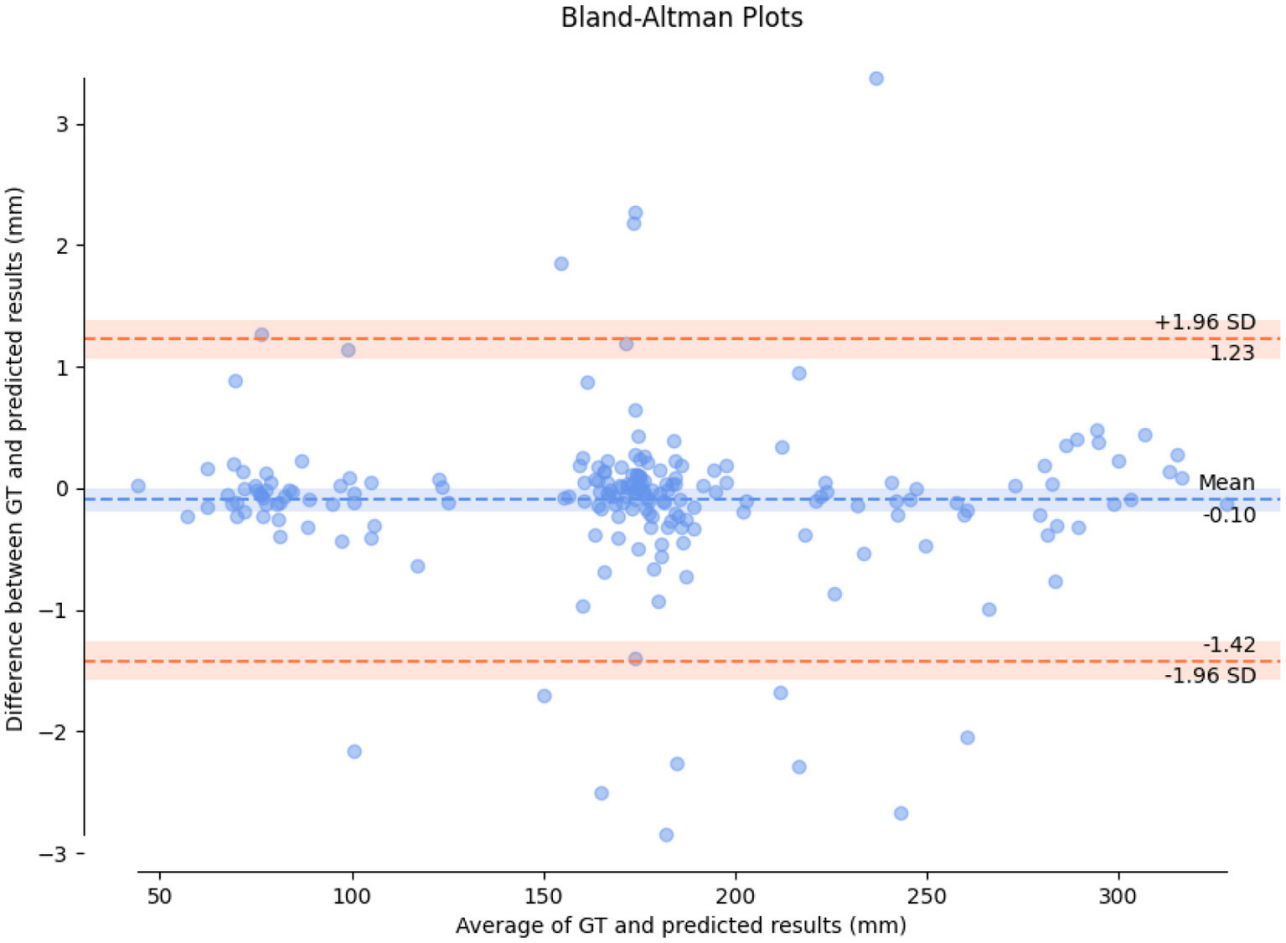
A Bland-Altman diagram on validation set.

In order to evaluate the consistency between HC measured by the proposed method and real value of HC, we draw a Bland-Altman diagram on validation set, as shown in Figure 8. Compared with the real value of HC, the Mean Difference of HC measurement is −0.10 mm with a 95% confidence interval and the error ranges from −1.42-1.23 mm. It indicated the HC measured by the proposed method has a good consistency with the real value.

## Conclusion

A new fetal head circumference auto-measurement method based on rotating ellipse detection has been proposed in this paper, which is a strictly end-to-end detection method without any post-processing for the task. As far as we know, this is the first application of end-to-end detection network to measure fetal head circumference directly. We combine transformer and CNN because convolution operations can extract rich context features in local area and transformer (MHSA) module can capture long-distance feature relationship benefitting from its ability of global and dynamic receptive fields. The two complement each other for promoting detection precision of fetal HC without significantly increasing the amount of computation. For the task of rotating elliptic object detection, the precision of angle regression is very important. Slight angle deviation will bring large changes in IOU. Therefore, we used SSR strategy for angle regression and added KLD that is approximate to IOU loss into total loss function. These methods significantly improve the detection precision. This study is expected to help less experienced sonographers, provide help for precision medicine, and relieve the shortage of sonographers for prenatal ultrasound in worldwide. There are also some shortcomings in our work, a little deviation can be allowed in predicting the location of target center point in the inference stage (that is, positive sample can be determined if the IOU is greater than a certain threshold), therefore, in order to facilitate calculation, we conducted pre-processing operation in the process of mapping the center point of ellipse to 2D Gaussian distribution on the heatmap. We generated the smallest horizontal enclosing rectangle of the ellipse, and used center point of rectangle as the new center point for mapping. There is a slight error with the center point of the ellipse, which may affect the precision of the detection results. This is also the study direction that we need to improve in the future.

## Data Availability Statement

Publicly available datasets were analyzed in this study. This data can be found at: https://hc18.grand-challenge.org.

## Author Contributions

CY: method and experiment design and manuscript writing. SL, ZY, and SY: data analysis and interpretation. JG, ZZ, YY, and YG: method investigation and experiment analysis. YK and CL: supervision. All authors contributed to the article and approved the submitted version.

## Funding

This study was supported by funding from the National Natural Science Foundation of China (grant numbers 81401143), National Key Research and Development Program of China (grant numbers 2018YFC1002900), the Stable Support Plan for Colleges and Universities in Shenzhen, China (grant numbers SZWD2021010), and the National Natural Science Foundation of China (grant numbers 62071311).

## Conflict of Interest

The authors declare that the research was conducted in the absence of any commercial or financial relationships that could be construed as a potential conflict of interest.

## Publisher's Note

All claims expressed in this article are solely those of the authors and do not necessarily represent those of their affiliated organizations, or those of the publisher, the editors and the reviewers. Any product that may be evaluated in this article, or claim that may be made by its manufacturer, is not guaranteed or endorsed by the publisher.

## Footnotes

^1^Available online at: https://hc18.grand-challenge.org.
